# Design of Driving Waveform for Shortening Response Time of Black Particles and White Particles in Three-Color Electrophoretic Displays

**DOI:** 10.3390/mi12111306

**Published:** 2021-10-25

**Authors:** Hu Zhang, Zichuan Yi, Simin Ma, Shaoning Deng, Weibiao Zhou, Wenjun Zeng, Liming Liu, Feng Chi, Yunfeng Hu, Chongfu Zhang, Li Wang, Jitao Zhang

**Affiliations:** 1 School of Electronic Science and Engineering (National Exemplary School of Microelectronics), University of Electronic Science and Technology of China, Chengdu 611731, China; 202021020727@std.uestc.edu.cn; 2 College of Eletron and Information，University of Electronic Science and Technology of China，Zhongshan Institute，Zhongshan 528402，China; masimin0402@163.com (S.M.); dengshaoning1@163.com (S.D.); weibiaozhou@163.com (W.Z.); zwjcareer@163.com (W.Z.); liulmxps@126.com (L.L.); chifeng@semi.ac.cn (F.C.); shanhuyf@163.com (Y.H.); cfzhang@uestc.edu.cn (C.Z.); 3 School of Information Engineering, Zhongshan Polytechnic, Zhongshan 528400, China; creekxi@163.com; 4 School of Mechanical and Electrical Engineering, Zhongshan Polytechnic, Zhongshan 528400, China; zhangjt20211@163.com

**Keywords:** three-color electrophoretic displays, driving waveform, response time, black particle, white particle, reference grayscale

## Abstract

The shortage of color in traditional electrophoretic displays (EPDs) can be compensated by three-color EPDs. However, the response time of black particles and white particles is increased. A new driving waveform based on the principle of three-color EPDs and electrophoresis theory was proposed to shorten the response time of black particles and white particles. The proposed driving waveform consisted of an erasing stage, an activation stage, a red driving stage, and a white or a black driving stage. The activation stage was mainly optimized in this paper. Firstly, the motion characteristics of the particles were analyzed using Stokes law and electrophoresis theory. Secondly, an optimal high frequency oscillation voltage was tested in order to improve the activity of the particles. Then, the influence of oscillation period and oscillation times on the activation stage were analyzed for optimizing the reference grayscale. According to the luminance of pixels, an oscillation period of 30 ms and an oscillation time of 30 were determined. The experimental results showed that the response time of black particles was shortened by 45%, and the response time of white particles was shortened by 40% compared with a traditional driving waveform.

## 1. Introduction

As a kind of reflective display, EPD has the advantages of paper-like display characteristics, a light weight, and an extremely low power consumption [1,2,3,4,5]. It has been widely used as an electronic paper technology in recent years. As a result, many materials and driving waveforms were proposed to improve its display effect. The performance of EPDs were greatly improved so that video playback could be realized. But only black-white video can be achieved in traditional EPDs, which have two types of charged particles in microcapsules [6]. In order to solve the shortage of color in traditional EPDs, three-color EPDs have been proposed. With the addition of red particles, three-color EPDs can display more different colors [7,8,9,10,11]. However, compared with the traditional EPDs, three-color EPDs have more types of particles in microcapsules, and the distribution of particles is more complicated, which increases the response time of black particles and white particles [12,13].

The driving waveform is a voltage sequence, and an electric field generated by it can control the movement of particles in microcapsules [14]. Therefore, it is of great significance to reduce the response time by optimizing the driving waveform. The physical model of the electric field has been proposed, which lays a theoretical foundation for optimizing the driving waveform [15,16]. The traditional driving waveform consists of an erasing stage, an activation stage, a red driving stage, and a white or a black driving stage [17,18,19]. The erasing stage is used to erase the original grayscale and realize direct current (DC) balance [20,21,22,23]. However, the image may not be erased for a long time. The longer the residence time of particles, the lower the activity, which leads to the increase of response time. The activation stage is used to improve the activity and reduce the viscosity of particles. Therefore, the response time of black particles and white particles is greatly affected at this stage [24]. The red driving stage and the white or black driving stage are used to drive particles to display a new image. Many driving waveforms were proposed to shorten the response time. A driving waveform which could optimize the activation stage was proposed, an inflection point was used to increase the duration of particles activation, and the distance which particles move to the target grayscale was reduced [25]. The duration of the activation stage was effectively reduced by this method, but it did not follow the rule of DC balance, which can easily cause damage to EPDs [26,27]. The distance which particles move to the target grayscale can be obtained by the reference grayscale. The grayscale conversion between the black and the white could be replaced by a shorter grayscale path to reduce the distance of particle motion. Therefore, the response time can be effectively reduced by optimizing the grayscale conversion path [11]. Furthermore, the effect of particle materials on response time is also a research focus [28,29]. Porous silica nanoparticles and silica coated with ionic liquid polymer nanoparticles were proposed to shorten the response time, which improved the display effect and provided a new idea for optimizing EPDs [30,31].

In order to reduce the black particles and white particles response time in three-color EPDs, the motion characteristics of particles were analyzed by using Stokes law and electrophoresis theory [32], and the distance between black particles and white particles and a common electrode plate was shortened by optimizing the activation stage of three-color EPDs, which effectively shortens black particles and white particles response time.

## 2. Principle of Three-Color EPDs

**Figure 1:**
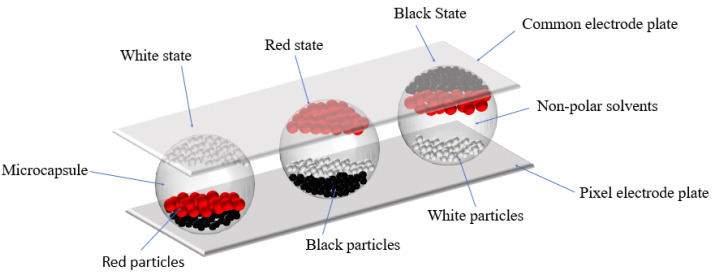
The structure of three three-color EPD pixels. When a negative voltage is applied to the pixel electrode plate, the white particles are driven to the common electrode plate and the pixel displays white, as shown on the left in this figure. When a low-amplitude positive voltage is applied to the pixel electrode plate, the red particles are driven to the common electrode plate and the pixel displays red, as shown on the middle in this figure. When a high-amplitude positive voltage is applied to the pixel electrode plate, the black particles are driven to the common electrode plate and the pixel displays black, as shown on the right in this figure.

The production of three-color EPDs is mainly based on microcapsule technology by filling three kinds of particles in each microcapsule. Each pixel is composed of non-polar solvents, a common electrode plate, a pixel electrode plate, negatively charged white particles, positively charged black particles, and red particles. The structure of three-color EPDs is shown in Figure 1. The particles are controlled by a driving waveform. Therefore, three-color EPDs can display different colors by applying different voltage sequences.

The display effect of three-color EPDs is determined by the movement of particles in microcapsules. It is of great significance to analyze the motion state of the particles. The motion state of these three kinds of particles are mainly affected by gravity, buoyancy, electric field force, and the Stokes force of non-polar solvent. In order to prevent the sedimentation of the particles, the density of the non-polar solvent is equal to the density of the particles. Therefore, the motion state of the particles is determined by the electric field force and the Stokes force. The resultant force of the electric field force and Stokes force is used to drive the particles, as shown in Equation (1).

(1) $$F=\frac{qU}{d}-6\pi \eta \nu R=m\frac{dv}{dt}$$

where F is the resultant force. q is the amount charge of the particle. U is the voltage applied to the pixel electrode plate. d is the distance between two electrode plates. η is the liquid viscosity coefficient. v is the velocity of the particle. R is the sphere radius. m is the mass of a particle. The first term in middle is the electric field force and the second term in middle is the Stokes force. $\frac{dv}{dt}$ is the acceleration of particles. The initial condition of the particles are static. The velocity of particles can be obtained by solving the differential Equation (1), as shown in Equation (2).

(2) $$\nu =\frac{qU}{6\pi d\eta R}\left(1-e^{-\frac{6\pi \eta R}{m}t}\right)$$

It can be seen that the velocity of particles is proportional to the voltage applied to the pixel electrode plate. The higher the applied voltage, the faster the particles’ velocity and the shorter the response time. However, excessive voltage would cause damage to three-color EPDs. The voltage in the white or black driving stage set to ±15 V. s is the distance between particles and the common electrode plate. The relationship between the black particles and white particles response time and s can be obtained based on Equation (2), as shown in Equation (3).

(3) $$s=\int_{0}^{T_{s}}vdt=\frac{qU}{6\pi d\eta R}\left(T_{s}+\frac{m}{6\pi \eta R}e^{-\frac{6\pi \eta R}{m}T_{s}}\right)-\frac{qUm}{36\pi^{2}\eta^{2}R^{2}d}$$

where $T_{s}$ is the black particles and white particles response time. It can be seen from Equation (3) that the black particles and white particles response time is proportional to the distance between particles and the common electrode plate. Thus, a shorter response time can be achieved by shortening s. s is related to the luminance of pixels. The luminance is inversely proportional to the distance between white particles and the common electrode plate and proportional to the distance between black particles and the common electrode plate. Therefore, s can be characterized by the luminance of pixels.

## 3. Experimental Results and Discussion

### 3.1. Experimental Platform

**Figure 2:**
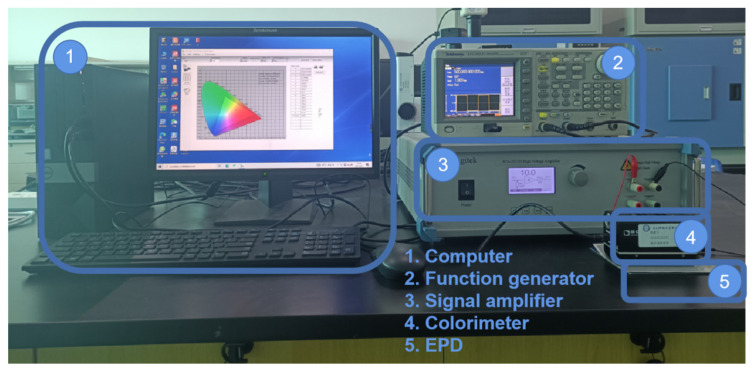
Experimental platform for the testing of EPD color gamut, luminance and response time. It was composed of a driving system and a testing system. (1) computer. (2) function generator. (3) signal amplifier. (4) colorimeter. (5) EPD.

The performance of three-color EPDs can be obtained by testing luminance and response time. In order to test and record luminance and response time during the driving process, an experimental platform was built, as shown in Figure 2. The driving system consisted of a computer (H430, Lenovo, Beijing, China), a function generator (AFG3022C, Tektronix, Beaverton, OR, USA), and a signal amplifier (ATA-2022H, Agitek, Xian, China), which were used to generate driving waveforms. The testing system consisted of a computer and a colorimeter (Arges-45, Admesy, Ittervoort, The Netherlands), which were used to collect and record experimental data, and a three-color EPD was used as an experimental object. It was designed by us and made by foundry (Dalian Longning Technology Co. Ltd., Dalian, China).

In the testing process, the driving waveform was edited by Arbexpress software in the computer. Then, the edited driving waveform was imported into the function generator through universal serial bus (USB). The voltage amplitude was amplified by the signal amplifier and then applied to the three-color EPD. Next, the luminance data was collected using a colorimeter and transmitted to the computer through USB. Finally, the luminance variation curve data was recorded in real-time using Admesy software.

### 3.2. Design of Driving Waveform

**Figure 3:**
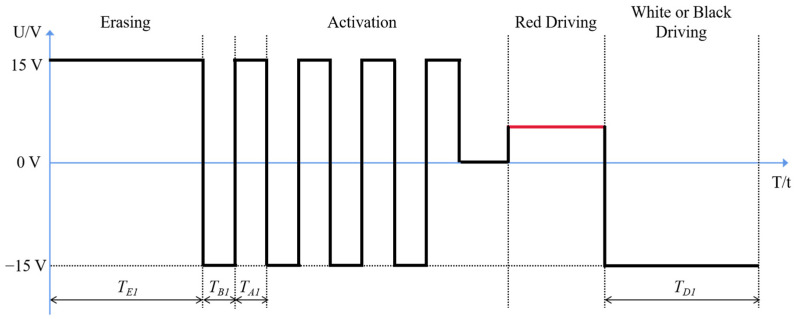
The traditional driving waveform of three-color EPDs. It consisted of an erasing stage, an activation stage, a red driving stage, and a white or a black driving stage. $T_{E1}$ is the duration of the erasing stage. $T_{B1}$ is the duration of negative voltage in one oscillation cycle, and $T_{A1}$ is the duration of positive voltage in one oscillation cycle. $T_{D1}$ is the duration of the white or black driving stage.

**Figure 4:**
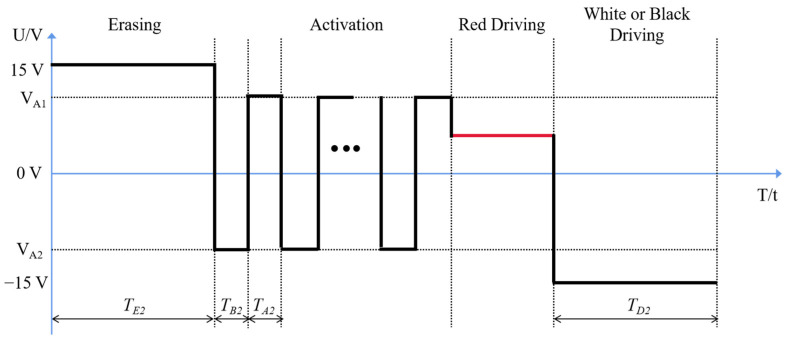
The proposed driving waveform in this paper. It consisted of an erasing stage, an activation stage, a red driving stage, and a white or a black driving stage. $T_{E2}$ is the duration of the erasing stage. $T_{B2}$ is the duration of negative voltage in one oscillation cycle, and $T_{A2}$ is the duration of positive voltage in one oscillation cycle. $T_{D2}$ is the duration of the white or black driving stage. $V_{A1}$ is the positive voltage of high frequency voltage value. $V_{A2}$ is the negative voltage of high frequency voltage value.

The traditional driving waveform of EPDs includes four stages: an erasing stage, an activation stage, a red driving stage, and a white or a black driving stage. As shown in Figure 3, after the activation stage of traditional driving waveform, the activity of particles is insufficient, which causes a long driving time. Therefore, a driving waveform based on the optimized activation stage was proposed to shorten response time and improve luminance performance, as shown in Figure 4.

In the activation stage, the proposed driving waveform is an improved high frequency oscillation waveform. $V_{A1}$ and $V_{A2}$ can be adjusted to maximize particle activity. The appropriate $T_{B2}$ and $T_{A2}$ are set to comply the rule of DC balance, and it was determined by Equation (4). The erasing stage of the proposed driving waveform is also used to erase the original image and less time is consumed due to optimization of the activation stage. Due to the position of particles and the target grayscale is shortened by the activation stage, the duration of the white or black driving stage of the proposed driving waveform is also shortened. In order to achieve DC balance, the erasing stage has the same duration as the white or black driving stage of the previous driving waveform, and the voltage polarity is opposite, as shown in Equation (5).

(4) $$V_{A1}\times T_{A2}+T_{A2}\times T_{B2}=0$$

(5) $$V_{E}\times T_{E2}+V_{D}{}^{1}\times T_{D}{}^{1}=0$$

where $V_{E}$ is the voltage of the erasing stage. $V_{D}{}^{1}$ is the voltage of the white or black driving stage of the previous driving waveform. $T_{D}{}^{1}$ is the duration of the white or black driving stage of the previous driving waveform. Since three-color EPD has a broader prospect, a red driving stage is designed to drive the red particles. The voltage sequence at this stage is designed according to the different threshold voltages of red particles and black particles [23].

### 3.3. Voltage Optimization in the Activation Stage

**Figure 5:**
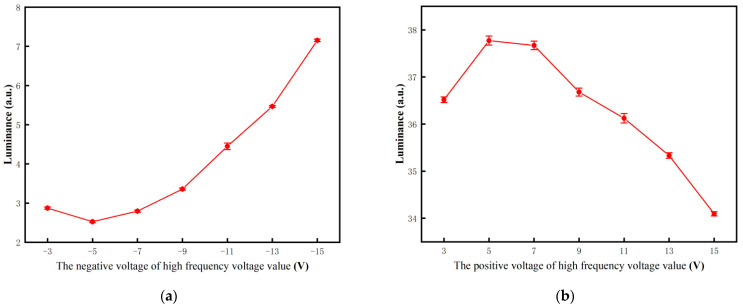
The relationship between luminance and activation voltage. (**a**) The luminance when it was driven by different $V_{A2}s$ when $V_{A1}$ was set to 15 V. The luminance was decreased with the decrease of $V_{A2}$ from −3 to −5 V, and it was increased with the decrease of $V_{A2}$ from −5 to −15 V. The minimum luminance of the three-color EPD was obtained when $V_{A2}$ was −5 V. (**b**) The luminance when it was driven by different $V_{A1}s$ when $V_{A2}$ was set to −15 V. The luminance was increased with the increase of $V_{A1}$ from 3 to 5 V, and it was decreased with the increase of $V_{A1}$ from 5 to 15 V. The maximum luminance of the three-color EPD was obtained when $V_{A1}$ was 5 V.

In order to obtain the optimal activation voltage for black particles, the effects of different $V_{A2}s$ on black particles were tested. The erasing stage and the white or black driving stage were set to 20 ms. $V_{A1}$ was set to 15 V. The oscillation period was set to 30 ms, and the oscillation times was set to 30. The luminance of the three-color EPD driven by different $V_{A2}s$ are shown in Figure 5a. It can be seen that the luminance was decreased with the decrease of $V_{A2}$ from −3 to −5 V, and it was increased with the decrease of $V_{A2}$ from −5 to −15 V. This was because the negative voltage was not enough to drive white particles when $V_{A2}$ was greater than −5 V, and black particles can be driven to the common electrode plate by $V_{A1}$. When $V_{A2}$ was less than −5 V, some white particles were mixed with black particles due to an increase of electrostatic force on white particles. The minimum luminance of the three-color EPD was obtained when $V_{A2}$ was −5 V. Therefore, the optimal activation voltage for black particles $V_{A2}$ was set to −5 V. Similarly, the effects of different $V_{A1}s$ on white particles were also tested, as shown in Figure 5b. The optimal activation voltage for white particles $V_{A1}$ was set to 5 V.

### 3.4. Oscillation Period and Oscillation Time Optimization in the Activation Stage

**Figure 6:**
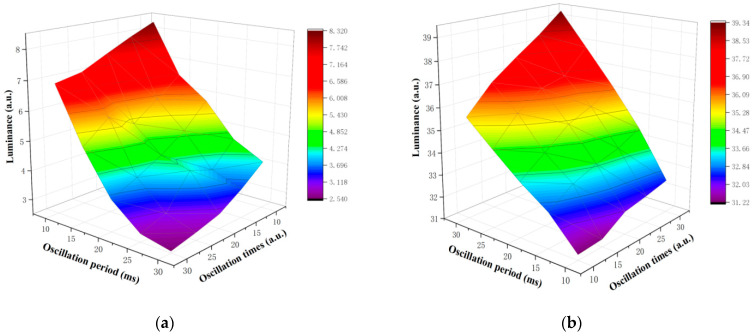
The erasing stage and the white or black driving stage were set to 20 ms and the high frequency oscillation voltage value was set to ±5 V. (**a**) The influence of oscillation period and oscillation times in luminance when the target grayscale was black. (**b**) The influence of oscillation period and oscillation times in luminance when the target grayscale was white.

In order to shorten the distance between particles and the common electrode plate, it is necessary to determine the optimal oscillation period and oscillation times by experiments. The erasing stage and the white or black driving stage were set to 20 ms and the high frequency oscillation voltage value was set to ±5 V. The oscillation period was set to 10 ms, 15 ms, 20 ms, 25 ms, and 30 ms, and the oscillation times were set to 10, 15, 20, 25, and 30. The luminance was measured in different oscillation periods and oscillation times. The experimental results are shown in Figure 6. Figure 6a shows the influence of oscillation period and oscillation times in luminance when the target grayscale was black, and Figure 6b shows the influence of oscillation period and oscillation times in luminance when the target grayscale was white. It can be seen that the luminance decreased with the increase of the oscillation period and oscillation times when the target grayscale was black, and the luminance increased with the increase of the oscillation period and oscillation times when the target grayscale was white. When the oscillation period was 30 ms and the oscillation time was 30, the luminance reached the minimum value of 2.55 when the target grayscale was black, and the luminance reached the maximum value of 39.33 when the target grayscale was white. It can be seen that black particles and white particles can be separated by high frequency oscillation voltage. However, the luminance could saturate as the oscillation period and oscillation times were increased, and the response time was increased due to more oscillation period and oscillation times. Therefore, the oscillation period was set to 30 ms and the oscillation times was set to 30 in the proposed driving waveform.

### 3.5. Response Time of the New Driving Waveform

**Figure 7:**
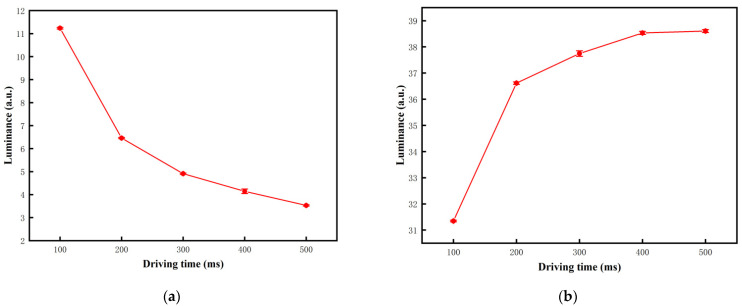
The relationship between luminance and the duration of white or black driving stage. (**a**) The luminance when the target grayscale was black and the minimum luminance was 3.53 when the duration was 500 ms. (**b**) The luminance when the target grayscale was white and the maximum luminance was 38.6 when the duration was 500 ms.

In order to compare the performance of traditional driving waveform and the proposed driving waveform, it is necessary to analyze the duration of each stage of traditional driving waveform. The oscillation period of the traditional driving waveform was set to 225 ms, and the oscillation times of the driving traditional waveform was set to 4, which can keep the same duration in activation stage. The white or black driving stage of the traditional driving waveform was set to 100–500 ms for testing the response time of the black and white particles. The luminance driven by the different duration of the white or black driving stage are shown in Figure 7. It can be seen that the luminance reached the minimum value of 3.53 when the target grayscale was black, and the luminance reached the maximum value of 38.6 when the target grayscale was white. It can be illustrated that the luminance could be saturated with the increase of the duration of white or black driving stage, which caused the response time of black particles and white particles to be increased. Therefore, the white or black driving stage of the traditional driving waveform was set to 500 ms.

**Figure 8:**
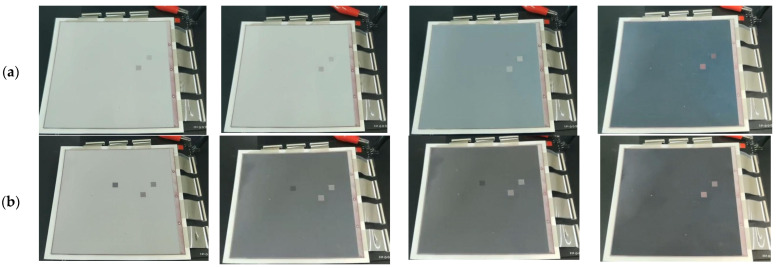

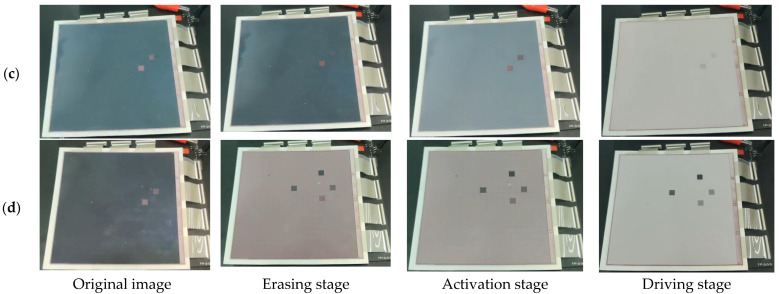
The grayscale conversion process of the three-color EPD. (**a**) It was driven by the traditional driving waveform and the target grayscale was black. (**b**) It was driven by the proposed driving waveform and the target grayscale was black. (**c**) It was driven by the traditional driving waveform and the target grayscale was white. (**d**) It was driven by the proposed driving waveform and the target grayscale was white.

**Figure 9:**
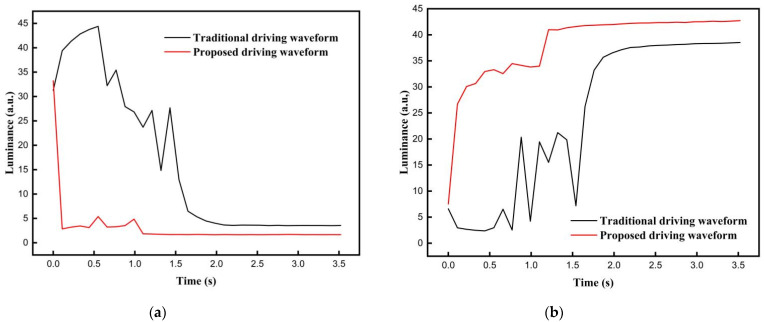
Luminance linear comparison of response time between the traditional driving waveform and the proposed driving waveform. (**a**) The relationship between luminance and response time when the target grayscale was black. (**b**) The relationship between luminance and response time when the target grayscale was white.

In addition, the grayscale conversion process of the three-color EPD driven by the traditional driving waveform and the proposed driving waveform is shown in Figure 8, and the number of flickers can be seen in Figure 9. Black curves represent the luminance curves of the traditional driving waveform. It can be seen that luminance curves were changed many times due to the fact that the frequency of the voltage in the activation stage was low. The number of flickers was nine and the maximum intensity was 44.38 when the target grayscale was black; the number of flickers was ten and the maximum intensity was 21.23 when the target grayscale was white. Red curves represent the luminance curves of the proposed driving waveform. It can be seen that there were fewer peaks in red curves. The number of flickers was three and the maximum intensity was 5.38 when the target grayscale was black; the number of flickers was two and the maximum intensity was 34.46 when the target grayscale was white. Therefore, compared with the traditional driving waveform, the number of flickers was reduced by 66.7% and the maximum intensity was reduced by 39 when the target grayscale was black; the number of flickers was reduced by 80% and the maximum intensity was reduced by 13.23 when the target grayscale was white.

Furthermore, the response time of black particles and white particles driven by the traditional driving waveform was compared with that of the proposed driving waveform. When the traditional driving waveform was applied to three-color EPDs, it can be seen that the luminance was stable at 3.5 when the target grayscale was black, and response time of black particles was 2.2 s; the luminance was stable at 38.6 when the target grayscale was white, and response time of white particles was 2.2 s. This was due to the long duration of the erasing stage and the activation stage, which prolonged the response time of black particles and white particles. When the proposed driving waveform was applied to three-color EPDs, it could be seen that the luminance was stable at 1.68 when the target grayscale was black, and response time of black particles was 1.21 s; the luminance was stable at 42.41 when the target grayscale was white, and response time of white particles was 1.32 s. This phenomenon proved that the improved activation stage can effectively shorten the distance between particles and the common electrode plate. Thus, the response time of black particles and white particles was shortened. Compared with the traditional driving waveform, the response time of black particles was shortened by 45%, the response time of white particles was shortened by 40%, the luminance was optimized by 52% when the target grayscale was black, and the luminance was optimized by 9.87% when the target grayscale was white.

## 4. Conclusions

In this paper, a new driving waveform based on electrophoresis theory was proposed to shorten the response time of black particles and white particles in three-color EPDs. The distance from particles to the common electrode plate was effectively reduced by optimizing the activation stage, which caused the response time of black particles and white particles to be decreased. Additionally, the colorimeter cannot detect some flickers due to the high voltage frequency of the activation stage. Therefore, the flickers were also reduced. Finally, compared with the traditional driving waveform, the shorter response time of particles and the better luminance performance can be obtained by using the proposed driving waveform, which provided a certain reference value for the application and development of three-color EPDs.
